# Survey evidence on public support for AI safety oversight

**DOI:** 10.1038/s41598-024-82977-5

**Published:** 2024-12-28

**Authors:** Stepan Vesely, Byungdoo Kim

**Affiliations:** 1 Department of Psychology, Norwegian University of Science and Technology, Trondheim, Norway; 2 Centre for Sustainable Business, King’s College London, London, United Kingdom

**Keywords:** AI safety, Regulation, Policy support, Risk preferences, Time preferences, Altruism, Human behaviour, Information technology

## Abstract

A number of AI safety concerns are being increasingly discussed by experts, including misinformation, invasion of privacy, job displacement, and criminal misuse. Two exploratory studies conducted in Germany and Spain (combined *n* = 2864) provide evidence that the general public largely supports strict oversight over safety of commercial artificial intelligence research. Among the factors that are associated with preferences for strict oversight are age, anticipated job displacement, innovativeness, and risk, time and altruistic preferences.

## Introduction

The development of artificial intelligence (AI) tools is on a rapidly increasing trajectory. However, advanced AI brings with it also new risks and may exacerbate existing concerns, including surveillance and invasion of privacy^1^, misinformation and manipulation^2^, discrimination^3^, liability and accountability concerns^4^, social risks related to disruptions of labor markets^5^, cybersecurity threats^6^, terrorism and other forms of criminal misuse^7^, and autonomous warfare^8^.

Regulatory oversight over safety of AI research and deployment could help mitigate some of these risks. Regulatory oversight may include risk disclosure and audit requirements^9^, standards and restrictions^10^, and in some cases outright bans^8^. Regulatory oversight may be usefully complemented by other policy instruments, such as taxes^11^, and by industry norms^12^.

Data from two large surveys we conducted in Germany and Spain indicate there is considerable appetite for “much stricter” regulatory oversight of commercial AI research, which is supported or strongly supported by 62.2 and 63.5% of the German and Spanish participants, respectively. We explore whether socio-economic characteristics, beliefs about future economic impacts of AI, innovativeness, altruism, risk preferences, and time preferences predict support for AI regulation.

## Method

### Participants and procedure

We report exploratory analyses of data that are part of two larger studies. Study 1 (Spanish residents, *n* = 1434) and Study 2 (German residents, *n* = 1430) were conducted in September 2023 with quota samples recruited by a market research company (SurveyEngine GmbH) from their partner panels. The surveys were programmed by SurveyEngine and hosted on their online platform. Sample socio-demographics can be found in Table 1.

The studies received approval from SIKT (Norwegian Agency for Shared Services in Education and Research, reference no. 345930). Our institution did not require an additional approval for the studies. The studies were conducted according to the principles expressed in the Declaration of Helsinki, as well as with ethics guidelines by the American Psychological Association and with national and institutional regulations. Written informed consent was provided by all participants.

**Table 1:** Sample socio-demographics.

Variable	Category	Percent of sample	
		Study 1 (*n* = 1434)	Study 2 (*n* = 1430)
Gender	Female	44.14	37.62
	Male	54.81	61.89
	Non-binary, other	0.35	0.28
	Missing	0.70	0.21
Age	Under 30 years of age	22.80	11.61
	30–39 years old	19.11	17.27
	40–49 years old	19.74	15.59
	50–59 years old	16.88	21.54
	Over 60 years of age	20.15	33.57
	Missing	1.32	0.42
Education	Mandatory education	2.16	14.76
	Some high school	3.28	29.65
	Graduated high school	25.52	21.12
	Some college	16.88	3.99
	University degree	49.79	29.02
	Missing	2.37	1.47
Yearly income before tax	Less than 15,000 EUR	16.39	12.59
	15,000–25,000 EUR	25.59	16.50
	25,000–35,000 EUR	26.50	20.49
	More than 35,000 EUR	23.71	43.22
	Missing	7.81	7.20

### Measures

*Support for AI safety oversight* is our dependent variable, measured with the item “Do you support much stricter regulatory oversight over safety of commercial artificial intelligence research?”, with response options “strongly oppose” (coded as 1), “somewhat oppose” (2), “neither support, nor oppose” (3), “somewhat support” (4), and “strongly support” (coded as 5).

We explore several possible determinants of support for AI oversight. The first group of predictors are participants’ expectations regarding AI’s economic impacts in a 10-year horizon. *Anticipated economic well-being* was measured with the item “What will in your estimate be the most likely impact of artificial intelligence on your economic well-being in 10 years from now?”, with response options “large reduction” (coded as 1), “moderate reduction” (2), “small reduction” (3), “no change” (4), “small improvement” (5), “moderate improvement” (6), and “large improvement” (coded as 7). *Anticipated job displacement* was measured with the item “What percentage of workers in your occupation will in your estimate be replaced by artificial intelligence within the next 10 years?”, with the following response options: “0–10%” (coded as 1), “10–30%” (2), “30–50%” (3), and “more than 50%” (coded as 4).

We measured participants’ innovativeness using two items adopted from Hurt et al.^13^: “I have to see other people use new inventions before I consider using them myself” and “I am skeptical to new inventions”, with response options “strongly disagree” (coded as 1), “somewhat disagree” (2), “neither agree, nor disagree” (3), “somewhat agree” (4), and “strongly agree” (coded as 5). The two items were not sufficiently correlated to form a reliable scale (*r* = 0.35 in Study 1, *r* = 0.41 in Study 2) and were therefore used as separate measures in the analysis, labelled *laggard* (the first item) and *skeptical* (the second item). Note that responses are coded such that higher scores indicate being more of a laggard or more skeptical to innovations.

The third group of predictors are participants’ altruism, time preferences, and risk preferences. *Altruism* was measured with an item adapted from Falk et al.^14^: “How do you assess your willingness to share with others without expecting anything in return?”, with response options ranging from 1 = “completely unwilling to share” to 7 = “extremely willing to share” (only the two extreme response options were labelled). *Time preferences* were measured with an item adapted from Falk et al.^14^: “In comparison to others, are you a person who is generally willing to give up something today in order to benefit from that in the future or are you not willing to do so?”, with response options ranging from 1 = “completely unwilling to give up something today” to 7 = “extremely willing to give up something today” (only the two extreme response options were labelled). *General risk preferences* were measured with an item adapted from Falk et al.^14^: “Are you a person who is generally willing to take risks or do you try to avoid taking risks?”, with response options ranging from 1 = “completely unwilling to take risks” to 7 = “extremely willing to take risks” (only the two extreme response options were labelled). *Economic risk preferences* were measured with an item based on Eckel and Grossman^15^. Participants were asked to select one of eight hypothetical lotteries with different levels of risk (see Table 2). Selection of lotteries was coded such that higher-risk lotteries received a higher score.

**Table 2:** Response options for the economic risk preferences item.

	50% chance of winning	50% chance of winning	I would pick:
Lottery 1	24 EUR	24 EUR	[Coded as 1]
Lottery 2	21 EUR	30 EUR	[Coded as 2]
Lottery 3	19 EUR	34 EUR	[Coded as 3]
Lottery 4	17 EUR	38 EUR	[Coded as 4]
Lottery 5	15 EUR	42 EUR	[Coded as 5]
Lottery 6	11 EUR	50 EUR	[Coded as 6]
Lottery 7	7 EUR	54 EUR	[Coded as 7]
Lottery 8	1 EUR	57 EUR	[Coded as 8]

The final group of predictors are participants’ socio-economic characteristics, including *gender*, *age*, *education*, *income*, and *household size*. Participants in addition stated their occupation by filling in a text field, but it was not possible to reliably categorize participants’ responses and this data was therefore excluded from analysis.

## Results

### Descriptive analysis

**Fig. 1:**
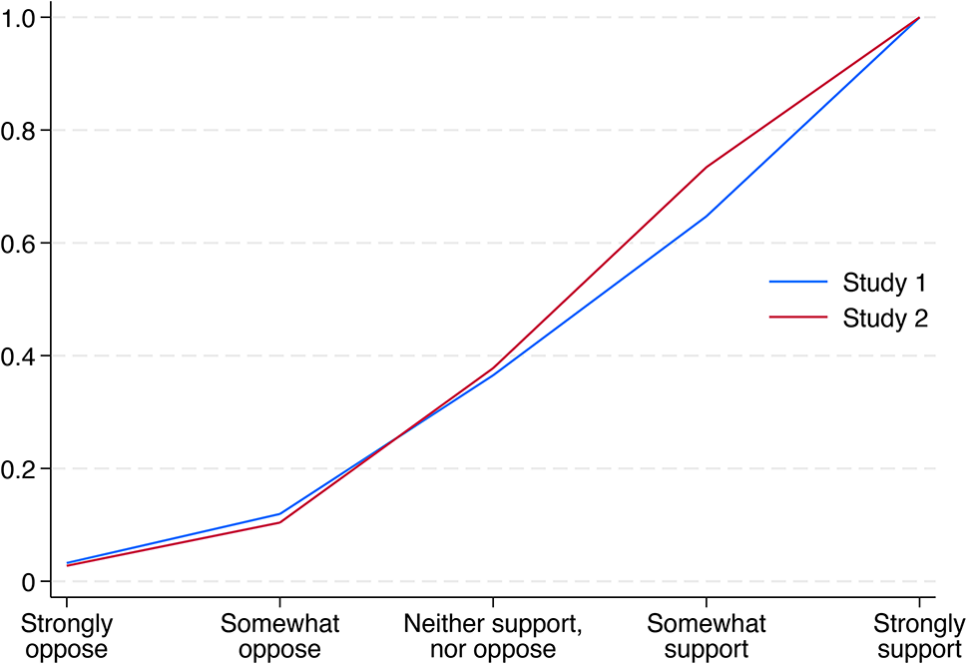
Cumulative distributions of support for AI safety oversight.

Figure 1 displays cumulative distributions of support for AI safety oversight in Studies 1 and 2. As can be seen from the figure, AI safety oversight enjoys similar levels of support in both countries. Only around 10% of participants oppose stricter AI safety oversight (11.9% of the Spanish participants in Study 1 and 10.4% of the German participants in Study 2). Around a quarter of participants do not have a preference either way (24.6% in Spain and 27.4% in Germany). The remaining nearly two thirds of participants somewhat support or strongly support much stricter AI safety oversight (63.5% in Spain and 62.2% in Germany).

Table 3 presents a breakdown of support for AI safety oversight by gender and age. Recall that a score of 4 indicates the participant “somewhat supports” strict AI oversight and a score of 3 indicates they “neither support, nor oppose” such policy. The majority of means reported in Table 3 fall within this range. While there are essentially no differences between men and women, participants reporting non-binary or other gender are descriptively speaking less supportive of AI safety oversight. Support for AI safety oversight tends to gradually increase with age, with about half a standard deviation change in support between the youngest and oldest age group. These patterns are formally tested in regression analyses reported below.

**Table 3:** Means and standard deviations of support for AI safety oversight by gender and age.

Variable	Category	Study 1 – mean (*SD*)	Study 2 – mean (*SD*)
Gender	Female	3.87 (1.07)	3.84 (1.03)
	Male	3.82 (1.13)	3.71 (1.00)
	Non-binary, other	3.20 (1.10)	1.75 (0.96)
Age	Under 30 years of age	3.58 (1.17)	3.49 (1.03)
	30–39 years old	3.78 (1.08)	3.40 (0.95)
	40–49 years old	3.75 (1.07)	3.67 (1.02)
	50–59 years old	4.03 (1.00)	3.82 (0.99)
	Over 60 years of age	4.09 (1.07)	4.03 (0.98)

### Regression analysis

Table 4 presents linear regression estimates, reporting standardized regression coefficients with 95% confidence intervals based on robust standard errors. We report *p*-values adjusted for multiple testing using the Benjamini and Hochberg method^16^. Collinearity is not an issue in either model (mean VIF = 1.17 in Study 1, mean VIF = 1.20 in Study 2).

**Table 4:** Determinants of support for AI safety oversight.

	Study 1 – Spain	Study 2 – Germany
	Std. coef. (95% CIs)	Std. coef. (95% CIs)
Anticipated economic well-being	0.02 (−0.04, 0.08)	−0.04 (−0.11, 0.02)
Anticipated job displacement	−0.07 (−0.14, −0.01)	−0.11** (−0.18, −0.05)
Laggard	0.15*** (0.09, 0.21)	0.12** (0.05, 0.18)
Skeptical	−0.11** (−0.17, −0.04)	0.06 (−0.01, 0.13)
Altruism	0.13*** (0.07, 0.19)	0.11** (0.04, 0.18)
Time preferences	0.02 (−0.04, 0.08)	0.11** (0.04, 0.17)
General risk preferences	−0.13*** (–0.20, –0.07)	−0.10** (−0.17, −0.04)
Economic risk preferences	−0.06 (−0.12, –0.01)	0.00 (−0.06, 0.06)
Male (vs. female)	−0.03 (−0.15, 0.08)	-0.01 (−0.14, 0.11)
Non-binary (vs. female)	−0.68* (−1.15, −0.21)	−1.70*** (−2.37, −1.03)
Age	0.08* (0.02, 0.15)	0.14*** (0.08, 0.20)
Education	0.05 (−0.01, 0.11)	0.01 (−0.05, 0.07)
Income	0.00 (−0.06, 0.06)	0.02 (−0.04, 0.08)
Household size	0.00 (−0.06, 0.06)	−0.02 (−0.08, 0.03)
Constant	0.02 (−0.06, 0.11)	0.02 (−0.09, 0.12)
Observations	1162	1093
*R* ^2^	0.104	0.130

*p < 0.05, **p < 0.01, *** *p* < 0.001. The gender variable has not been standardized, and so estimates for “male” and “non-binary” indicate how these categories differ from “female” in terms of the standardized dependent variable.

Respondents expecting AI to lead to more job displacement in their profession are less supportive of AI safety regulation (only marginally statistically significant in the Spanish sample). Bivariate correlations between anticipated job displacement and support for AI oversight are also negative, *r* = −0.16 in both studies. An explanation of this finding is not obvious, nevertheless it seems to speak against a motivated reasoning account which would be more consistent with a positive correlation between the two variables (wherein workers fearing job displacement could attempt to slow down AI progress through demanding strict regulation).

As expected, laggards and, at least in the German sample, those skeptical to new inventions are more supportive of AI safety regulation. Surprisingly, skepticism appears to be associated with less support for regulation in the Spanish sample – this is the least intuitive finding in our two studies and should be interpreted with caution.

The analysis also suggests that social and economic preferences may play an important role in shaping preferences for AI safety regulation. Altruistic, risk averse, and – in the German case – patient participants are more supportive of strict AI safety oversight.

Finally, older participants are more supportive and participants who identify as non-binary are less supportive of strict AI safety regulation (the latter finding is based on a small number of cases, five in Spain and four in Germany, and as potentially unreliable is therefore not considered in the following discussion). There do not appear to be other socio-demographic drivers of regulation support.

Looking at statistically significant predictors’ standardized beta coefficients, we see from Table 4 that a one standard deviation increase in a predictor is associated with between 8 and 15% of a standard deviation change in support for AI safety oversight. Unreported eta-squared statistics show that these predictors individually explain between 0.6% (age in Study 1) and 2.0% (laggard in Study 1) of variance in the dependent variable. We consider predictors uniquely explaining less than 1.0% of variance in the dependent variable to be of negligible importance and predictors explaining between 1.0 and 2.0% of variance to be of modest importance. From this perspective, of the statistically significant predictors, the importance of age would be considered negligible in the Spanish sample (uniquely explaining 0.6% of variance in the dependent variable) but modestly important in the German sample (uniquely explaining 1.8% of variance in the dependent variable). The importance of general risk preferences would be considered negligible in the Spanish sample (uniquely explaining 0.9% of variance in the dependent variable) but again modestly important in the German sample (uniquely explaining 1.5% of variance in the dependent variable). In summary, all statistically significant predictors in Table 4 show some degree of relevance.

Importantly, also because of the relatively weak correlations between predictors (|0.01| < *r* < |0.37| in Study 1 and |0.01| < *r* < |0.46| in Study 2, excluding non-binary participants), the models as a whole explain considerable amounts of variance in support for AI safety oversight – 10.4% in the Spanish sample and 13.0% in the German sample.

## Concluding remarks

We report result from, to our knowledge, the first two studies on the general population’s preferences for AI safety oversight. The majority of respondents were in favor of strict safety oversight. Interestingly, socio-demographic factors only played a minor role (similarly see König et al.^17^). Less innovative participants were more supportive of safety oversight, largely as expected (although the results were somewhat mixed).

Participants anticipating greater job displacement due to AI over the next ten years were less supportive of AI safety oversight. This finding goes against the intuition that people concerned about job displacement may want to politically leverage demands for stricter AI oversight to slow down AI progress and its presumed labor market impacts with it. We should caution that the effect was small and only statistically significant in Germany (marginally so in Spain). Nevertheless, it may warrant further exploration in subsequent research. Jeffrey^18^ similarly found that making economic vulnerability to AI and automation more salient decreased support for certain welfare policies. One possible explanation for our finding is that participants might view strict AI safety regulation as a facilitator, not of AI research directly, but of commercial adoption of the now better regulated and more trusted AI tools. Another possibility is that participants concerned about job displacement could be primarily focused on legislation addressing this problem directly and may even oppose legislation that they feel could de-emphasize their policy priorities (see Gallego et al.^19^). These interpretations involve different assumptions about the electorate’s political and technological foresight. We therefore suggest that an analysis of the role played by foresight in shaping preferences for policies responding to large technological shifts could be promising.

Our results also reveal a connection between risk, time and altruistic preferences and support for AI safety oversight. An emerging literature similarly suggests associations between economic and social preferences and technology acceptance^20^. Subsequent research on AI safety regulation and AI regulation more broadly can build on the present findings by further examining the role of people’s economic and social preferences in this domain for example by manipulating perceptions of AI risk and of who, when and with what probability may be impacted by it.

The theoretical approach taken in this paper is purposefully eclectic given the highly exploratory nature of the present studies and lack of previous research on the topic. We focus on correlations with basic socio-economic variables and beliefs about the technology’s economic impact, with key economic preferences which have been shown to play a role in the broader technology adoption literature^20^ , and with consumer innovativeness. Subsequent research can build on this work by replicating our findings concerning risk, time and altruistic preferences, innovativeness, and socio-economic factors, as well as by incorporating other theoretical perspectives, such as influences postulated in the Unified Theory of Acceptance and Use of Technology^21^. Additional economic and social preferences, such as preferences for fairness and economic efficiency, should also be considered. For example, a recent study^22^ found that acceptance of AI-powered facial recognition tools, an AI-application associated with potential privacy risks, is predicted by perceptions of fairness. Given the novel nature of AI technologies and their potential broad social impacts, it could be beneficial to incorporate a wide range of influences when attempting to understand people’s preferences for whether and how the technology should be regulated.

### Limitations

Measures collected for the purposes of this paper were part of longer questionnaires, and so simplicity and ease of comprehension were a priority. A downside of this approach was that single-item measures were used to operationalize most constructs, potentially leading to effect size attenuation due to measurement error. Our estimates may therefore be interpreted as lower bounds of the true associations. Where possible we relied on widely used measures, and where no widely used measures were available we constructed our own. Development of psychometrically validated scales to measure vulnerability to AI, in particular, could be a useful direction for subsequent research (besides items introduced in this paper see also other recent work^18,19^).

Subsequent research may not only consider inclusion of multi-item measures of relevant constructs, but also consider measuring different facets of the broader constructs. We took a first step in this direction by including a measure of general risk preferences^14^ and a measure of economic risk preferences^15^, anticipating that these could play different roles in predicting support for AI safety oversight. Indeed, only general risk preferences predicted support for AI safety oversight.

Finally, even though some effects replicated across studies (associations with innovativeness, altruism, risk preferences, and age), subsequent confirmatory research is needed to further substantiate the present findings.

## Data Availability

Data used in this paper are available from the corresponding author upon request.

## References

[1] Beraja, M., Kao, A., Yang, D. Y. & Yuchtman, N. AI-tocracy. *Q. J. Econ.* **138**, 1349–1402 (2023).

[2] Somoray, K. & Miller, D. J. Providing detection strategies to improve human detection of deepfakes: an experimental study. *Comput. Hum. Behav.* **149**, 107917 (2023).

[3] Obermeyer, Z., Powers, B., Vogeli, C. & Mullainathan, S. Dissecting racial bias in an algorithm used to manage the health of populations. *Science* **366**, 447–453 (2019). 31649194 10.1126/science.aax2342

[4] Hacker, P. The European AI liability directives – critique of a half-hearted approach and lessons for the future. *Comput. Law Secur. Rev.* **51**, 105871 (2023).

[5] Berg, A., Buffie, A. F. & Zanna, L. F. Should we fear the robot revolution? (the correct answer is yes). *J. Monet. Econ.* **97**, 117–148 (2018).

[6] Kaloudi, E. & Li, J. The AI-based cyber threat landscape: a survey. *ACM Comput. Surveys* **53**, 20 (2020).

[7] Urbina, F., Lentzos, F., Invernizzi, C. & Ekins, S. Dual use of artificial-intelligence-powered drug discovery. *Nat. Mach. Intell.* **4**, 189–191 (2022). 36211133 10.1038/s42256-022-00465-9 PMC9544280

[8] Russell, S. AI weapons: Russia’s war in Ukraine shows why the world must enact a ban. *Nature* **614**, 620–623 (2023). 36810886 10.1038/d41586-023-00511-5

[9] Falco, G. et al. Governing AI safety through independent audits. *Nat. Mach. Intell.* **3**, 566–571 (2021).

[10] Vokinger, K. N. & Gasser, U. Regulating AI in medicine in the United States and Europe. *Nat. Mach. Intell.* **3**, 738–739 (2021). 34604702 10.1038/s42256-021-00386-z PMC7611759

[11] Acemoglu, D. & Lensman, T. Regulating transformative technologies. Working paper. (2023).

[12] Srikumar, M. et al. Advancing ethics review practices in AI research. *Nat. Mach. Intell.* **4**, 1061–1064 (2022).

[13] Hurt, H. Y., Joseph, K. & Cook, C. D. Scales for the measurement of innovativeness. *Hum. Commun. Res.* **4**, 58–65 (1977).

[14] Falk, A., Becker, A., Dohmen, T., Huffman, D. & Sunde, U. The preference survey module: a validated instrument for measuring risk, time, and social preferences. *Manag. Sci.* **69**, 1935–1950 (2023).

[15] Eckel, C. & Grossman, P. J. Sex differences and statistical stereotyping in attitudes toward financial risk. *Evol. Hum. Behav.* **23**, 281–295 (2002).

[16] Benjamini, Y. & Hochberg, Y. Controlling the false discovery rate: a practical and powerful approach to multiple testing. *J. Royal Stat. Soc. Ser. B* **57**, 289–300 (1995).

[17] König, P. D., Wurster, S. & Siewert, M. B. Sustainability challenges of artificial intelligence and citizens’ regulatory preferences. *Government Inform. Q.* 101863. (2023).

[18] Jeffrey, K. Automation and the future of work: how rhetoric shapes the response in policy preferences. *J. Econ. Behav. Organ.* **192**, 417–433 (2021).

[19] Gallego, A., Kuo, A., Manzano, D. & Fernández-Albertos, J. Technological risk and policy preferences. *Comp. Polit. Stud.* **55**, 60–92 (2022).

[20] Schleich, J., Gassmann, X., Meissner, T. & Faure, C. A large-scale test of the effects of time discounting, risk aversion, loss aversion, and present bias on household adoption of energy-efficient technologies. *Energy Econ.* **80**, 377–393 (2019).

[21] Venkatesh, V., Morris, M. G., Davis, G. B. & Davis, F. D. User acceptance of information technology: toward a unified view. *MIS Q.* **27**, 425–478 (2003).

[22] Li, R. G. Institutional trustworthiness on public attitudes toward facial recognition technology: evidence from U.S. policing. *Gov. Inform. Q.* **41**, 101941 (2024).

